# Scabies prevalence after ivermectin-based mass drug administration for lymphatic filariasis, Samoa 2018–2019

**DOI:** 10.1371/journal.pntd.0011549

**Published:** 2023-08-22

**Authors:** Gabriela A. Willis, Therese Kearns, Helen J. Mayfield, Sarah Sheridan, Robert Thomsen, Take Naseri, Michael C. David, Daniel Engelman, Andrew C. Steer, Patricia M. Graves, Colleen L. Lau

**Affiliations:** 1 Research School of Population Health, Australian National University, Canberra, Australia; 2 Menzies School of Health Research, Charles Darwin University, Darwin, Australia; 3 School of Public Health, Faculty of Medicine, University of Queensland, Brisbane, Australia; 4 National Centre for Immunisation Research and Surveillance, Sydney, Australia; 5 Ministry of Health, Apia, Samoa; 6 School of Medicine and Dentistry, Griffith University, Gold Coast, Australia; 7 The Daffodil Centre, The University of Sydney, a joint venture with Cancer Council NSW, Sydney, Australia; 8 Tropical Diseases, Murdoch Children’s Research Institute, Melbourne, Australia; 9 College of Public Health, Medical and Veterinary Sciences, James Cook University, Cairns, Australia; Hebrew University Hadassah Medical School, ISRAEL

## Abstract

**Background:**

Scabies is a common skin infestation caused by the *Sarcoptes scabei* mite. Ivermectin, one of three drugs used in mass drug administration (MDA) for lymphatic filariasis, is also effective for treating scabies. Ivermectin-based MDA was first conducted in Samoa in August 2018, with ivermectin being offered to those aged ≥5 years. Here, we report scabies prevalence in Samoa after MDA.

**Methods:**

We conducted household surveys 1.5–3.5 months (Survey 1) and 6–8 months (Survey 2) after the 2018 MDA in 35 primary sampling units. We conducted clinical examination for scabies-like rash and used International Alliance for the Control of Scabies classification criteria. We estimated scabies prevalence by age, gender and region. Multivariable logistic regression was used to assess factors associated with prevalence.

**Results:**

We surveyed 2868 people (499 households) and 2796 people (544 households) aged 0–75 years in Surveys 1 and 2, respectively. Scabies prevalence increased from 2.4% (95% CI 2.1–2.7%) to 4.4% (95% CI 4.0–4.9%) between surveys. Scabies was associated with younger age (0–4 years: aOR 3.5 [2.9–4.2]; 5–15 years: aOR 1.6 [1.4–1.8] compared to ≥16 years), female gender (aOR 1.2 [95% CI 1.1–1.4]; region (aOR range from 1.4 [1.1–1.7] to 2.5 [2.1–3.1] between regions), large households (aOR 2.6 [2.0–3.4] households ≥13), and not taking MDA in 2018 (aOR 1.3 [95% CI 1.1–1.6]).

**Conclusions:**

We found moderate prevalence of scabies in two population-representative surveys conducted within 8 months of the 2018 MDA for lymphatic filariasis. Prevalence appeared to increase between the surveys, and ongoing surveillance is recommended, particularly in young children.

## Author summary

Scabies is a common skin disease that is caused by a small mite that burrows into the skin. It can be treated by a drug called ivermectin, which was given to every person aged 5 years and over in Samoa in 2018 to treat a different disease, known as lymphatic filariasis. We wanted to understand how common scabies was in the people of Samoa and so did two surveys where we visited people’s houses that we randomly selected. We asked people in each household questions about scabies symptoms and looked at their skin for signs of scabies. We surveyed 2868 people in 499 households in the first survey and 2796 in 544 households in the second survey. We found that 2.4% of people in the first survey and 4.4% of people in the second survey had scabies. We also found that some people were more likely to have scabies: younger children, women, people living in some regions of Samoa, people living with more people in their household, and people who didn’t take the medicine for lymphatic filariasis in 2018.

## Introduction

Scabies is a common neglected tropical disease, with up to 450 million incident cases per year globally, with the highest prevalence in the Pacific Islands and Latin America [1–3]. Scabies is caused by infestation with the microscopic mite *Sarcoptes scabiei var*. *hominis* and is predominantly transmitted through close personal contact [4]. The mite burrows into the skin and consumes the epidermis and sera, triggering an immune response causing rash and intense itching [5]. Cases are asymptomatic in the first four to six weeks after initial infestation, and most cases are diagnosed clinically when they present with rash and itching [6–8]. Scabies affects all age groups but is more common in children and in low- and middle-income countries [2]. Overcrowding is a driver of transmission, and institutional outbreaks occur in settings such as schools, prisons, aged care facilities, and hospitals [2].

Itching from scabies can be debilitating, and the direct effects on the skin lead to 0.21% of disability-adjusted life-years worldwide [9]. Additionally, scabies lesions can become secondarily infected with *Streptococcus pyogenes* and/or *Staphylococcus aureus* [4,10,11], and potentially result in a substantial disease burden. *S*. *pyogenes* pyoderma has been associated with acute post-streptococcal glomerulonephritis [4,12] and there is evidence to suggest an association with acute rheumatic fever [13,14].

Mass drug administration (MDA) with ivermectin has been shown to effectively reduce scabies in several studies, with varying success in sustaining reduction in prevalence [3,15–18]. Trials from Pacific Island countries with very high scabies prevalence have shown a 90% reduction in scabies using ivermectin-based MDA. In these trials, oral ivermectin was given to most of the population, and topical permethrin to groups where ivermectin is currently contra-indicated [17,19]. Reductions in scabies prevalence were sustained for 24 and 36 months respectively [20,21]. However, other studies from Tanzania and northern Australia, with lower baseline prevalence, did not demonstrate a sustained reduction [15,22]. However, a difference in the drug regimen may have influenced findings in the Tanzanian study, with only one dose of ivermectin being given annually and those under 5 years of age being offered benzyl benzoate cream [22], while a high proportion of new entries to the cohort was thought to have influenced findings in the Australian study [15].

Recently, a framework for scabies control was developed after an informal WHO consultation [23]. The framework recommended that where prevalence is 10% or above, two doses of ivermectin-based MDA given 7–14 days apart can be considered as a method for public health control. Three to five annual rounds are proposed, with a minimum coverage target of 80% of the population [23].

Despite the high global burden of scabies, global prevalence estimates have largely been based on modelling studies, with limited robust data from surveillance systems or field surveys [23,24]. In the Western Pacific region, school-based and household surveys from several countries, including Samoa, have reported high prevalence [25–28]. In Samoa, a survey in 1997 found scabies prevalence of 4.9% (95% confidence interval [CI] 3.0–7.0%) [29]; the authors raised the possibility that the lower than expected prevalence might be the result of ivermectin-containing MDA, which was used for lymphatic filariasis control in Samoa in 1996 and 1997 [30]. In February 2018, a school-based survey conducted in Samoa (localised areas in the southern parts of Upolu island) found scabies prevalence of 14.4% among children aged 4–15 years [28]. In neighbouring American Samoa, a review of medical records in 2011–2012 estimated an annual incidence of presentation with scabies of 29.3 per 1,000 among children aged ≤14 years (99.9 per 1,000 in children aged less than one year), with bacterial superinfection in 53% of cases [31].

In August 2018, the Samoa Ministry of Health implemented the first round of MDA for lymphatic filariasis using three drugs (ivermectin, diethylcarbamazine, and albendazole) for eligible adults and children aged ≥5 years, while children aged 2–4 years were offered diethylcarbamazine and albendazole only [32]. In this paper, we report on scabies prevalence in Samoa in two population representative surveys of all ages conducted within an eight month period after the 2018 MDA, and we investigated risk factors for infection.

## Methods

### Background and setting

Samoa is an independent country in the South Pacific with a population of approximately 200,000 [33]. Most of the population live on the two main islands of Upolu (where the capital city, Apia, is located) and Savai’i. Samoa is divided into four administrative regions: Apia Urban Area (20% of population), Northwest Upolu (35% of population), Rest of Upolu (23% of population), and Savai’i (22% of population). There are approximately 338 villages, with an average population of 580 (range <20 to 4300) per village [34].

The Surveillance and Monitoring to Eliminate Lymphatic Filariasis and Scabies (SaMELFS) study in Samoa was conducted with the primary aim of assessing the impact of MDA on lymphatic filariasis and scabies [32,35,36]. Due to logistic reasons, the first survey was delayed and took place 1.5 to 3.5 months after MDA (September to November 2018, henceforth “Survey 1”), instead of prior to MDA as intended. The second survey was conducted 6 to 8 months after the MDA (March to May 2019, henceforth “Survey 2”). The SaMELFS surveys presented an opportunity to assess scabies prevalence in all age groups in the months following nationwide ivermectin-based MDA.

### Ethics

Ethical approval was obtained from human research ethics committees at the Samoa Ministry of Health and The Australian National University (protocol 2018/341). The study was conducted in collaboration with the Samoa Department of Health, World Health Organization Samoa country office, Samoa Red Cross, and The Task Force for Global Health. Written informed consent was obtained from all participants, or parent/guardian in the case of minors aged <18 years.

### Study design

Detailed description of the sampling design has been previously reported [35,37]. In brief, participants were surveyed from 35 primary sampling units (PSUs) across Upolu, Savai’i, and Manono Islands. Each PSU consisted of one large or two smaller villages, and a total of 43 individual villages were included. Fifteen households were selected from each PSU, and household members of all ages were invited to participate in the scabies survey. If nobody was home at the time of the visit, the house was revisited later in the day and/or on another day. If household members were still absent at the second attempt and the target sample size had not yet been met, the household was replaced with the nearest inhabited household. The same PSUs were selected for both surveys. Households were selected independently for each survey, and by chance, a small number of households were included in both surveys. For lymphatic filariasis, convenience surveys of children aged 5–9 years were also conducted outside of the home, e.g., at schools and community centres. However, scabies examination was not conducted during convenience surveys because of privacy reasons.

### Sample size calculation

A target sample size of 2000 people in each age group (5–9 years, and ≥10 years) was required to detect a critical threshold of 2% filarial antigen prevalence, with a 5% chance of type 1 error, 75% power (when true prevalence is 1%), a design effect of 2.0, and correcting for the finite population [35]. Scabies prevalence was anticipated to be approximately 10%, so the above samples size was assumed to be adequate for estimating prevalence. Household members of all ages (including those aged <5 years) were invited to participate in the scabies survey.

### Training of field teams

A five-day training program (including two days of pilot village surveys) was conducted before each survey and included didactic and practical sessions on study background and rationale, field logistics, obtaining informed consent, and field data collection using smartphones. Field teams were trained in clinical examination for scabies based on training programs used in other studies [38,39], and provided with pictorial flipcharts to use during surveys. The lead trainer was an Australian nurse with experience in scabies field surveys (TK) and was available through both surveys to assist field teams. The same training methods and materials were used prior to both surveys.

Field team members consisted of community workers from the Samoa Red Cross, staff from the Ministry of Health, Samoan student nurses (2019), and Australian researchers (nurse, doctors, epidemiologists). Each field team included at least one clinician (nurse or student nurse), with remote support by the Australian nurse or doctors when required, including telephone discussion and review of de-identified photographs of rashes. Some team members participated in both surveys.

### Enrolment

We contacted community leaders (mayors, pastors, chiefs) in each village to discuss the survey and arrange suitable dates and times for the field team to visit and followed up with a hand delivered letters to confirm details. The village leaders informed the households about the timing and purpose of the survey. On the day of the survey, households were approached by a Samoan team member to obtain verbal consent to discuss survey participation with the family. If the head of the household agreed to participate, individual written informed consent was obtained from those eligible. An individual was eligible to participate if the selected house was their primary place of residence, and/or if they slept there the previous night.

### Data collection

Questionnaire and clinical examination data were collected electronically using Secure Data Kit software (www.securedatakit.com, now Standard Data) on smartphones, and electronic records were uploaded and stored on a SQL secure cloud-based server. Each participant was assigned a unique scannable QR code to link their enrolment/consent form, questionnaires, and blood samples.

### Questionnaires

Questionnaire data were collected through interviews with participants or their parent/guardian. Interviews were conducted in Samoan or English, depending on the participant’s preference. Questions included information on demographics, occupation, number of household members, and participation in the 2018 MDA. Scabies-specific questions included skin itching, scratching, or scabies-like rash experienced by the participants, their household members, and close contacts (other family, friends, classmates).

### Clinical examination for scabies

Exposed skin on arms and legs was examined to identify the presence and distribution of scabies-like lesions. In children, the abdomen, back and chest were also examined. The field teams recorded data on the rash appearance (typical scabies, atypical scabies, or other rash), and distribution of the lesions.

### Scabies classification and definitions

Scabies was defined using three categories within the International Alliance for the Control of Scabies (IACS) Criteria (Table 1) [40]. Classification was completed during data analysis into the following categories: no scabies, B3, C1, C2, and ‘*any scabies’* (combination of B3, C1 and C2).

**Table 1:** Definitions of scabies categories, typical distribution, and history features according to IACS Criteria [41].

	Classification	Description
**Scabies categories**	Clinical–B3 Suspected–C1 Suspected–C2 ‘*Any scabies’*	Typical lesions in a typical distribution and two history features Typical lesions in a typical distribution and one history feature Atypical lesions or atypical distribution and two history features Any of B3, C1, or C2 combined
**Typical Distribution**	All ages Aged <2 years	Lesions on the hands/wrists/arms or feet/ankles/lower legs Above, plus lesions on the face/head/neck or abdomen/chest/back
**History Features**	H1 H2	Itch Positive contact history, defined as one or more of the following: • Close contact with an individual diagnosed with scabies • Close contact with an individual with itch that is not accounted for by another condition

IACS = International Alliance for the Control of Scabies.

### Data analysis

Data analysis was conducted using Stata version 15 (Statacorp 2017). Descriptive analyses were used to report the demographic characteristics of participants. Crude scabies prevalence by year, region, age groups, and gender were calculated, and the Clopper-Pearson binomial method was used to estimate 95% confidence intervals (CI). Prevalence estimates were adjusted for survey design using Stata command *svyset (*adjusting for probability of PSU selection within the country, probability of household selection within PSU, clustering at PSU and household, and finite population correction over all PSUs and households; standard errors were calculated using Taylor-linearised variance estimation). Prevalence estimates (using Stata command *proportion*) were standardised for age (5-year age groups from Samoa 2016 census) and gender.

Using *‘any scabies’* as the outcome measure, multivariable logistic regression (Stata command *svy*:*logistic*) was used to estimate odds ratios for covariates, including year, region, age group, gender, number of household members (as a proxy for crowding), at least one employed person in the household (as a proxy for socioeconomic status) and participation in 2018 MDA. Covariates that were significant at *p*<0.2 in univariable analysis were included in the final multivariable model. The intracluster correlation coefficient (ICC) was calculated using mixed-effects logistic regression (Stata commands *melogit* and *estat icc*) to provide a measure of how similar observations were within PSUs and households. Statistical significance was defined as *p*<0.05.

## Results

### Participants

At Survey 1, 2868 participants from 499 households were examined for scabies (median 5 persons per household, range 1–31). Survey 2 included a similar number of participants from the same PSUs (2796 participants from 544 households, median four persons per household, range 1–21). The number of participants by age group differed between the two surveys due to lower number of participants aged <5 years at Survey 2, however, the median age was similar between surveys (Table 2). There was no difference in gender distribution between the two surveys, nor the proportion of those aged ≥5 years who recalled taking MDA in 2018. In both surveys, almost all participants were born in Samoa (99%) and participants were similarly distributed between the four regions.

**Table 2:** Demographics of study population included in scabies Surveys 1 and 2, Samoa.

		Survey 1 n (%)	Survey 2 n (%)
**Survey timing**	Time since 2018 MDA*	1.5 to 3.5 months	6 to 8 months
**Household characteristics**	Number of households surveyed	499	544
	Participants per household (median, IQR)	5 (3–7)	4 (3–7)
	Residents per household (median, IQR)	8 (6–12)	8 (5–10)
**Participant characteristics**	Number of participants	2868	2796
	Age groups (years)		
	0–4^	479 (16.7)	188 (6.7)
	5–15	817 (28.5)	974 (34.8)
	≥16	1572 (54.8)	1634 (58.4)
	Median age (IQR)	19 (7–40)	20 (10–42)
	Gender		
	Male	1356 (47.3)	1254 (44.9)
	Female	1512 (52.7)	1542 (55.2)
	Region		
	Apia Urban Area	489 (17.1)	437 (15.6)
	North-West Upolu	1181 (41.2)	1113 (39.8)
	Rest of Upolu	665 (23.2)	638 (22.8)
	Savai’i	533 (18.6)	608 (21.8)
	Employed person surveyed in household		
	Yes	1225 (42.7)	1248 (44.6)
	No	1643 (57.3)	1548 (55.4)
	MDA in 2018^#^		
	Recalled swallowing tablets	2101 (87.9)	2319 (88.9)

*Ivermectin, diethylcarbamazine, and albendazole mass drug administration (MDA) was distributed nationally for lymphatic filariasis elimination in August 2018.

IQR = interquartile range.

^In Survey 1, 198 aged<2 years, 281 aged 2–4 years; in Survey 2, 60 aged <2 years, 128 aged 2–4 years.

^#^Only applicable to those aged ≥5 years (Survey 1 N = 2386, Survey 2 N = 2608).

### Scabies prevalence, and comparison between Survey 1 and 2

According to IACS classifications, 74 (2.6%, 95% CI 2.0–3.2%) and 103 (3.7%, 95% CI 3.0–4.5%) participants were diagnosed with scabies (B3, C1 or C2) in Survey 1 and Survey 2, respectively. Most of the cases identified were clinical scabies (B3). Scabies-like lesions (typical or atypical) were identified in 93 participants (3.2%, 95% CI 2.6–3.9%) in Survey 1, and 133 participants (4.8%, 95% CI 4.0–57%) in Survey 2 (Table 3). In Surveys 1 and 2, 81.0% and 75.2% of rashes were in a typical distribution for scabies, respectively. In Survey 1, 82.8% and 76.3% reported itch and positive contact history features, respectively, while in survey 2, 75.2% and 24.8% reported these features, respectively.

**Table 3:** Crude prevalence of scabies lesions and diagnosis by IACS classifications, Samoa, Surveys 1 and 2.

	Survey 1		Survey 2	
	N	Crude prevalence % (95% CI)	N	Crude prevalence % (95% CI)
**Number of participants**	2868		2796	
**Scabies Lesions**				
Typical	66	2.3 (1.8–2.9)	82	2.9 (2.3–3.6)
Atypical	27	0.9 (0.6–1.3)	51	1.8 (1.3–2.4)
Total	93	3.2 (2.6–3.9)	133	4.8 (4.0–5.7)
**Scabies prevalence by IACS classifications**				
B3 –Clinical	42	1.5 (1.1–2.0)	46	1.7 (1.2–2.2)
C1 –Suspected	11	0.4 (0.2–0.7)	15	0.5 (0.3–0.9)
C2 –Suspected atypical	21	0.7 (0.5–1.1)	42	1.5 (1.1–2.0)
‘Any scabies’ (B3, C1, & C2 combined)	74	2.6 (2.0–3.2)	103	3.7 (3.0–4.5)

IACS = International Alliance for the Control of Scabies

**Fig 1:**
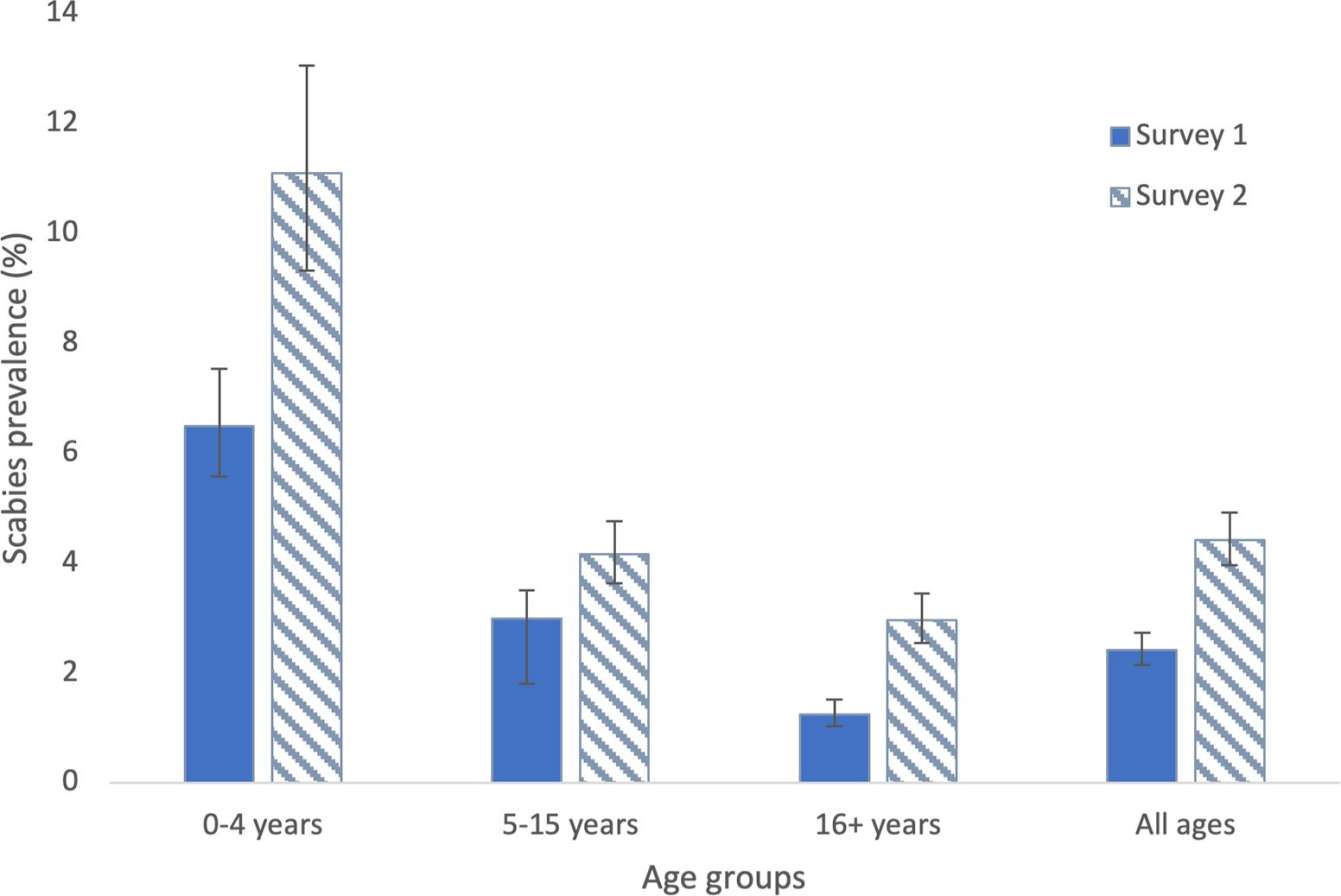
Prevalence of ‘any scabies’ (IACS B3, C1 and C2 classifications combined) by age groups, Survey 1 (solid bars) and Survey 2 (striped bars), Samoa. Prevalence estimates adjusted for survey design. Prevalence for ‘all ages’ standardised for age and gender, and results for age groups standardised for gender.

After adjusting for survey design and standardizing for age and gender, scabies prevalence was higher in Survey 2 (4.4%, 95% CI 4.0–4.9%) than in Survey 1 (2.4%, 95% CI 2.1–2.7%) (Table 4 and Fig 1), with a greater increase among females than males. Age-specific prevalence was highest among those aged 0–4 years, and higher in Survey 2 than Survey 1 for all age groups (Table 4 and Fig 1). In participants aged 0–4 years, adjusted prevalence of clinical scabies (B3) was higher in Survey 2 (7.3%, 95% CI 6.2–8.4%) than Survey 1 (3.5%, 95% CI 2.9–4.4%), but not significantly different for suspected scabies (C1 or C2). In those aged ≥16 years, clinical scabies (B3) was also more common in Survey 2 (3.0%, 95% CI 2.5–3.4%) than Survey 1 (1.2%, 95% CI 1.0–1.5%). Scabies prevalence by diagnostic category and age group for Surveys 1 and 2 are provided in S1 Table.

**Table 4:** Adjusted prevalence of ‘any scabies’ (IACS B3, C1 and C2 classifications combined) by gender and age, Samoa, Surveys 1 and 2.

	Survey 1	Survey 2
	Adjusted prevalence* **of ‘any scabies’ % (95% CI)**	Adjusted prevalence* **of ‘any scabies’ % (95% CI)**
**All participants** ^**a**^ ^,^ ^**b**^	2.4 (2.1–2.7)	4.4 (4.0–4.9)
**Gender** ^**a**^		
Male	2.4 (2.0–2.9)	3.5 (3.0–4.0)
Female	2.4 (2.1–2.9)	5.4 (4.8–6.0)
**Age groups (years)** ^**b**^		
0–4	6.5 (5.6–7.5)	11.1 (9.3–13.0)
5–15	3.0 (2.5–3.6)	4.2 (3.6–4.8)
≥16	1.2 (1.0–1.5)	3.0 (2.5–3.4)

IACS = International Alliance for the Control of Scabies. ‘Any scabies’ = IACS B3, C1 & C2 classifications combined.

*All categories adjusted for survey design (clustering and household selection probability within PSU). ^a^Standardised for age. ^b^Standardised for gender.

**Fig 2:**
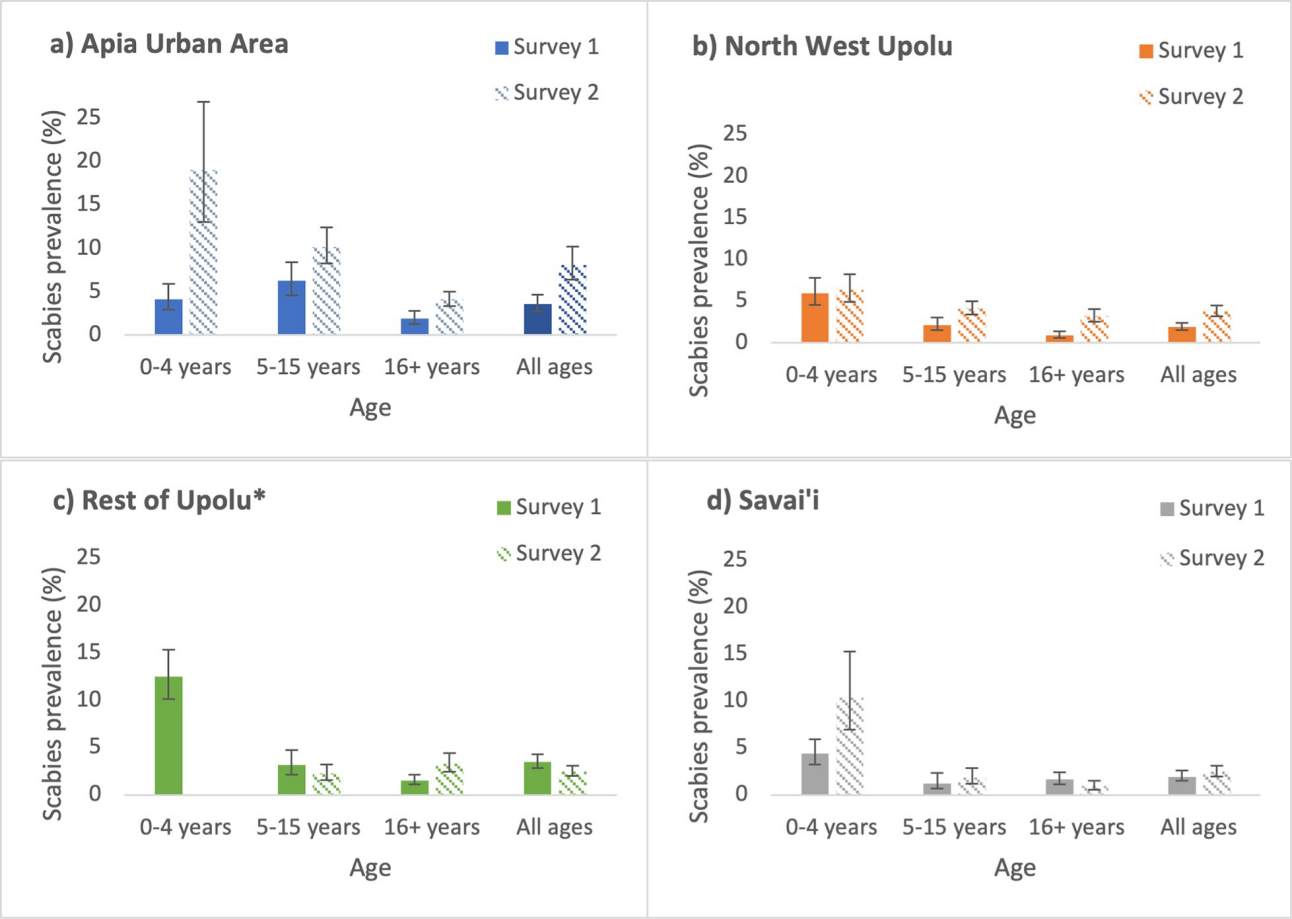
Prevalence of ‘any scabies’ (IACS B3, C1 and C2 classifications combined) by age group and region, Survey 1 (solid bars) and Survey 2 (striped bars), Samoa. **a) Apia Urban Area, b) North West Upolu, c) Rest of Upolu, and d) Savai’i.** Prevalence estimates adjusted for survey design and standardised for age and gender. (*Results not reported for participants aged 0–4 years in Rest of Upolu in Survey 2 because <5 participants were screened in this subgroup).

Adjusted prevalence for all ages was higher in Survey 2 than in Survey 1 for three of the four regions, and the difference was statistically significant for Apia Urban Area and North-West Upolu (Fig 2). There was a statistically significant increase in scabies prevalence between the surveys in the following age- and region-specific groups: 0–4 years and ≥16 years in Apia Urban Area; 5–15 years and ≥16 years in North-West Upolu; ≥16 years in Rest of Upolu; and 0–4 years in Savai’i (Fig 2).

### Clustering of scabies at PSU and household levels

There was evidence of clustering of scabies at the PSU level (ICC 0.53 and 0.32 in Surveys 1 and 2, respectively). Scabies prevalence by PSU for Surveys 1 and 2 are shown in S2 Table.

Clustering at the household level was more intense than at the PSU level (ICC 0.70 and 0.49 in Surveys 1 and 2, respectively).

### Risk factors for scabies

Among participants of all ages, after adjusting for Survey 1 or 2, region, household size, gender, age groups, at least one employed person screened at the household, participation in 2018 MDA, and survey design, significant risk factors for scabies were: surveyed in survey 2 (adjusted odds ratio [aOR] 2.1 (1.9–2.4); female gender (aOR 1.2 [95% CI 1.1–1.4]; age <16 years (aOR 3.4 [95% CI 2.9–4.2] for 0–4 years, and aOR 1.6 [95% CI 1.4–1.8] for 5–15 years compared to those aged ≥16 years); living in a large household (13 or more residents in household, aOR 2.6 [95% CI 2.0–3.4] compared to households with 1–4 residents); not taking MDA in 2018 (aOR 1.3 [95% CI 1.1–1.6]); and living in a region other than Savai’i, with the highest risk in Apia Urban Area (aOR 2.5, 95% 2.1–3.1) (Table 5). Findings were similar when only participants aged ≥5 years were included in the model (i.e., those eligible for ivermectin in 2018 MDA) (S3 Table).

**Table 5:** Crude (univariate model) and adjusted (multivariable model) odds ratios for prevalence of ‘any scabies’ (IACS B3, C1, and C2 classifications combined) among all ages, Samoa, Surveys 1 and 2.

	Sample size (N)	Scabies (n)	Adjusted prevalence* (95% CI)	Unadjusted Odds ratio (95% CI)	Adjusted Odds Ratio (95% CI)^	Multivariable model *p* value
**Total participants Survey**	5664	177	3.3 (3.0–3.6)	-	-	
Survey 1	2868	74	2.4 (2.1–2.7)	1.0	1·0	
Survey 2	2796	103	4.4 (4.0–4.9)	1.6 (1.4–1.9)	**2.1 (1.9–2.4)**	<0·001
**Region**						
Apia Urban Area	926	43	5.4 (4.6–6.2)	2.6 (2.1–3.2)	**2.5 (2.1–3.1)**	<0.001
North-West Upolu	2294	63	2.9 (2.5–3.3)	1.3 (1.1–1.6)	**1.4 (1.1–1.7)**	0.004
Rest of Upolu^#^	1303	43	4.1 (3.6–4.5)	1.6 (1.3–2.0)	**1.9 (1.5–2.3)**	<0.001
Savai’i	1141	28	2.2 (1.9–2.5)	1·0	1·0	-
**Number of residents in household**						
1–4	859	20	2.8 (2.2–3.5)	1.0	1·0	-
5–8	2323	60	2.7 (2.3–3.1)	1.5 (1.2–1.9)	**1.4 (1.1–1.8)**	0.01
9–12	1357	43	3.0 (2.6–3.4)	1.8 (1.5–2.3)	**1.7 (1.3–2.1)**	<0.001
13–20	1125	54	4.9 (4.3–5.5)	3.0 (2.4–3.8)	**2.6 (2.0–3.4)**	<0.001
**Gender**						
Male	2610	73	2.9 (2.6–3.2)	1.0	1.0	-
Female	3054	104	3.7 (3.3–4.2)	1.2 (1.0–1.4)	**1.2 (1.1–1.4)**	0·004
**Age groups (years)**					
0–4	667	54	7.9 (7.0–8.9)	4·0 (3.5–4.6)	**3.5 (2.9–4.2)**	<0·001
5–15	1791	60	3.6 (3.1–4.1)	1.7 (1.5–1.9)	**1.6 (1.4–1.8)**	<0·001
≥16	3206	63	2.1 (1.8–2.3)	1·0	1·0	-
**At least one employed person screened in the household**						
Yes	2473	74	3.3 (2.9–3.8)	1.0	1.0	-
No	3191	103	3.3 (2.9–3.8)	1.0 (0.8–1.2)	1.2 (1.0–1.4)	0.09
**Took ivermectin-based MDA in 2018** †						
Yes	4420	107	2.3 (2.1–2.6)	1.0	1·0	-
No	1244	70	4.3 (3.5–5.2)	2.4 (2.1–2.7)	**1.3 (1.1–1.6)**	0.004

*Adjusted for survey design (clustering and household selection probability within PSU) and standardized for age and gender

^Multivariable logistic regression model adjusted for survey design (sampling probability by PSU and household)

^#^ Logistic regression models included the children aged ≤5 years from Rest of Upolu (n = 3), even though prevalence was not reported for this group in Fig 2

† Includes participants aged 0–4 years not eligible for ivermectin-containing MDA

## Discussion

Our study found moderate prevalence (2.4% and 4.4%) of scabies among two population-representative surveys of all age groups in Samoa conducted within eight months of the 2018 ivermectin-based MDA for lymphatic filariasis. We found regional differences in scabies prevalence, and risk factors for infection, including young age (<16 years), female gender, and living in large households. Among those aged ≥5 years, non-participation in the MDA in 2018 was associated with higher scabies prevalence.

The overall prevalence of 2.4% and 4.4% in our two surveys were below the currently recommended threshold of 10% community prevalence for starting MDA for scabies [23,41]. Where the prevalence is moderate (2-<10%) the recommendations are less clear, due to lack of evidence. Our findings are similar those from a survey conducted in 1999 among Samoan children aged 5–17 years where scabies prevalence was 4.9% [29]; notably two rounds of ivermectin-containing MDA were distributed in 1996 and 1997 [30]. Our results were considerably lower than the 14.4% prevalence identified in a school-based survey of 4–15 year-olds conducted by Taiaroa *et al* in February 2018, but this study was limited to a few small communities in southern Upolu [28]. While the 2018 study was conducted prior to MDA, we were not able to use the results as a pre-MDA baseline because the survey was not nationally representative, and there may be considerable variation in scabies prevalence between regions and communities. Furthermore, Taiaroa *et al* used a different case definition for scabies that did not include a history of itch or household contacts with itch [28].

We found higher scabies prevalence in Survey 2 (6–8 months post-MDA) than in Survey 1 (1.5–3.5 months post-MDA). Overall prevalence in all age groups increased from 2.4% (95% CI 2.1–2.7%) to 4.4% (95% CI 4.0–4.9%). The greatest increase was observed among those aged under 5 years, from 6.5% (95% CI 5.6–7.5%) to 11.1% (9.3–13.1%), with most of the increase observed in category B3 (clinical scabies). While the increase in prevalence was statistically significant, the public health significance of this finding is unclear. The results were difficult to interpret because baseline prevalence immediately before MDA was not available and increase in prevalence could have been due to natural fluctuation in prevalence or random error. However, it is possible that this may represent reinfestation, particularly as young children <5 years of age were not treated with ivermectin and only one round of ivermectin-based MDA was distributed in Samoa prior to our surveys, less than the three to five rounds of annual ivermectin-based MDA recommended for scabies [23,41].

Our study identified highest prevalence among those aged under 5 years, who were not given ivermectin during the 2018 MDA. We also found higher prevalence in females, possibly because of closer and more frequent contact with young children through caring duties such as feeding and carrying. Higher prevalence among households with more residents is consistent with the general understanding that crowding is a risk factor for infestation [31]. More intense clustering of scabies at the household level compared to community level suggests the importance of transmission between household members. Among those aged over 5 years, higher prevalence in those who did not take MDA in 2018 (after adjusting for age, sex, and household size) suggests that ivermectin may have been effective in reducing prevalence, but other factors may be at play. A recent community-based trial in Fiji found that one dose of ivermectin-based MDA (with topical permethrin to contraindicated groups) was highly effective at reducing scabies prevalence (from 15.2% to 2.7% over 12 months) and non-inferior to two doses [42], but we were unable to assess the impact of ivermectin-containing MDA in Samoa because of the absence of a baseline assessment. The reasons for regional differences in scabies prevalence in Samoa are unclear, but notably the two regions with significantly higher overall prevalence in Survey 2 than Survey 1 (Apia Urban Area and North West Upolu) are the most densely populated, and also reported lower MDA coverage rates in 2018 than the other regions [32].

Our study has several limitations, perhaps the most significant being the absence of a baseline (pre-MDA) survey making interpretation of results challenging. As the survey methods were designed for the primary objectives relating to lymphatic filariasis, our surveys included a relatively low number of children aged under five years (479 in Survey 1 and 188 in Survey 2), potentially affecting the generalisability of the findings. Our proxy measure for socioeconomic status (at least one employed person screened in household) did not account for employed persons who were not home during the survey, or those who may have declined to participate. Impetigo data were not collected as we were measuring the indirect impact of LF ivermectin MDA on scabies prevalence.

Measurement bias may also have occurred as scabies examinations were mostly conducted by field workers who had limited clinical experience in scabies diagnosis. Despite supervision and remote support, this may have led to some misclassification. Additionally, different field teams between the two surveys might also have contributed to differences in results between years, but this issue was minimised by using the same training methods and materials immediately prior to each survey. In addition, it is likely the design effect was underestimated in our calculation of the study’s required sample size based on sample ICCs, therefore reducing the study’s effective sample size and reducing the study’s power. However, the impact of this underestimation does not appear to have had a substantial impact on precision estimates, as confidence intervals reported in Tables 3–5 or Figs 1 and 2 appear not to be unduly wide. Longer term follow-up surveys were planned but have been delayed because of prolonged border closures related to COVID-19 pandemic.

Despite the limitations, our study provides the largest population representative household survey of scabies in Samoa and one of the largest population surveys of scabies in the Pacific Islands. To minimise errors related to classifying scabies into IACS categories during fieldwork, we only asked field teams to record clinical observations (rash, distribution, number), and classifications were done during the data analysis phase. This approach greatly simplified fieldwork activities and should be considered during field surveys when clinical experts are not readily available.

Based on our results, ongoing surveillance is recommended for monitoring scabies prevalence in Samoa. Further increases in prevalence may justify targeted control strategies for scabies. Considering that scabies prevalence was highest among young children, more intensive surveillance in this age group may be warranted, potentially integrated with childhood programs (e.g., immunisation visits, well baby clinics) or school health team visits. Integration with other public health surveillance programs (e.g., for lymphatic filariasis and other neglected tropical diseases) would improve cost-effectiveness and long-term sustainability.

## Supporting information

S1 Table Crude and adjusted prevalence of scabies by age group and International Alliance for the Control of Scabies (IACS) classifications (41), Samoa, Surveys 1 and 2. (PDF)

S2 Table Crude and adjusted prevalence of scabies by PSU, Samoa, Surveys 1 and 2. (PDF)

S3 Table Crude (univariate model) and adjusted (multivariable model) odds ratios for scabies prevalence (IACS B3, C1 and C2 classifications combined) in participants aged ≥5 years (eligible for ivermectin in 2018 MDA), Samoa, Surveys 1 and 2. (PDF)
