# Supplementary material for: Scabies prevalence after ivermectin-based mass drug administration for lymphatic filariasis, Samoa 2018–2019
Source: PLoS Negl Trop Dis. 2023 Aug 22;17(8):e0011549. doi: 10.1371/journal.pntd.0011549 (PMC10497159; doi:10.1371/journal.pntd.0011549)
Supplement: S1 Table — (PDF) [file pntd.0011549.s001.pdf]

S1 Table. Crude and adjusted prevalence of scabies by age group and International Alliance for the Control of Scabies (IACS) classifications (41), Samoa, Surveys 1 and 2.

Age group (years)	IACS classifications	Survey 1			Survey 2		
		n	Crude prevalence % (95% CI)	Adjusted prevalence* % (95% CI)	n	Crude prevalence % (95% CI)	Adjusted prevalence* % (95% CI)
**0-4**	B3 – Clinical	22	4.6 (2.9-6.9)	3.5 (2.9-4.4)	14	7.4 (4.1-12.2)	7.2 (6.2-8.4)
	C1 – Suspected	3	0.6 (0.1-1.8)	0.7 (0.5-1.1)	2	1.1 (0.1-3.8)	1.0 (0.6-1.6)
	C2 – Suspected atypical	8	1.7 (0.7-3.3)	2.2 (1.6-3.1)	5	2.6 (0.9-6.1)	2.8 (2.0-3.8)
	'Any scabies' (B3, C1, & C2)	33	6.9 (4.8-9.5)	6.5 (5.6-7.5)	21	11.2 (7.0-16.6)	11.0 (9.3-13.0)
**5-15**	B3 – Clinical	10	1.2 (0.6-2.2)	1.1 (0.9-1.5)	13	1.3 (0.7-2.3)	1.5 (1.2-1.9)
	C1 – Suspected	3	0.4 (0.1-1.1)	0.2 (0.2-0.3)	5	0.5 (0.2-1.2)	0.6 (0.4-0.9)
	C2 – Suspected atypical	10	1.2 (0.6-2.2)	1.6 (1.2-2.1)	19	2.0 (1.2-3.0)	2.0 (1.7-2.4)
	'Any scabies' (B3, C1, & C2)	23	2.8 (1.8-4.2)	3.0 (2.4-3.6)	37	3.8 (2.7-5.2)	4.2 (3.6-4.8)
**≥16**	B3 – Clinical	10	0.6 (0.3-1.2)	0.7 (0.5-0.9)	19	1.2 (0.7-1.8)	1.6 (1.3-2.1)
	C1 – Suspected	5	0.3 (0.1-0.7)	0.5 (0.3-0.7)	8	0.5 (0.2-1.0)	0.3 (0.3-0.5)
	C2 – Suspected atypical	3	0.2 (0.0-0.6)	0.1 (0.1-0.2)	18	1.1 (0.6-1.7)	1.0 (0.8-1.2)
	'Any scabies' (B3, C1, & C2)	18	1.1 (0.7-1.8)	1.2 (1.0-1.5)	45	2.8 (2.0-3.7)	3.0 (2.5-3.4)

*Adjusted for survey design (clustering and household selection probability within PSU) and standardised for gender
