# Supplementary material for: Scabies prevalence after ivermectin-based mass drug administration for lymphatic filariasis, Samoa 2018–2019
Source: PLoS Negl Trop Dis. 2023 Aug 22;17(8):e0011549. doi: 10.1371/journal.pntd.0011549 (PMC10497159; doi:10.1371/journal.pntd.0011549)
Supplement: S2 Table — (PDF) [file pntd.0011549.s002.pdf]

S2 Table. Crude and adjusted prevalence of 'any scabies' by PSU, Samoa, Surveys 1 and 2

PSU	Survey 1			Survey 2		
	n	Crude prevalence % (95% CI)	Adjusted prevalence* % (95% CI)	n	Crude prevalence % (95% CI)	Adjusted prevalence* % (95% CI)
1	76	0.0 (0.0-4.7)	0 (-)	66	7.6 (2.5-16.8)	6.6 (5.7-7.6)
2	76	5.3 (1.5-12.9)	11.8 (10.9-12.7)	60	3.3 (0.4-11.5)	1.5 (1.2-1.9)
3	76	0.0 (0.0-4.7)	0 (-)	82	4.9 (1.3-12.0)	3.4 (2.5-4.5)
4	84	3.6 (0.7-10.1)	3.6 (2.7-4.6)	71	5.6 (1.6-13.8)	4.5 (3.5-5.8)
5	100	6.0 (2.2-12.6)	7.1 (6.4-7.8)	83	12.0 (5.9-21.0)	16.9 (15.5-18.4)
6	77	1.2 (0.0-7.0)	1.3 (0.9-1.9)	75	5.3 (14.7-13.1)	5.0 (4.3-5.8)
7	81	0.0 (0.0-4.4)	0 (-)	90	2.2 (2.7-7.8)	2.3 (1.7-3.2)
8	82	1.2 (0.0-6.6)	1.0 (0.7-1.3)	91	3.3 (0.7-9.3)	3.0 (2.2-3.9)
9	92	1.0 (0.0-5.9)	1.1 (0.7-1.6)	70	11.4 (5.1-21.3)	11.1 (9.8-12.5)
10	82	3.6 (0.8-10.3)	5.0 (3.9-6.4)	73	1.4 (0.0-7.4)	1.0 (0.7-1.3)
11	82	0.0 (0.0-0.4.3)	0.0 (-)	74	0 (0.0-4.9)	0.0 (-)
12	78	6.4 (2.1-14.3)	5.8 (4.6-7.4)	78	3.8 (0.8-10.8)	5.6 (4.8-6.5)
13	96	0.0 (0.0 -3.8)	0.0 (-)	77	7.8 (2.9-16.2)	8.9 (7.5-10.7)
14	85	0.0 (0.0-4.2)	0.0 (-)	86	0.0 (0.0-4.2)	0.0 (-)
15	84	0.0 (0.0-4.3)	0.0 (-)	69	1.4 (0.0-7.8)	1.3 (1.0-1.7)
16	85	0.0 (0.0-4.2)	0.0 (-)	69	2.9 (3.5-10.1)	2.3 (1.9-2.8)
17	90	4.4 (1.2-11.0)	3.1 (2.4-3.9)	75	0.0 (0.0-4.8)	0.0 (-)
18	78	0.0 (0.0-1.4)	0.0 (-)	78	5.1 (1.4-12.6)	2.5 (2.2-2.9)
19	74	4.0 (0.8-11.4)	9.3 (8.7-9.9)	107	7.5 (3.3-14.2)	7.7 (6.7-8.7)
20	92	4.3 (1.2-10.8)	3.3 (2.6-4.2)	76	5.3 (1.4-12.9)	5.4 (4.6-6.2)
21	84	2.4 (0.3-8.3)	1.8 (1.4-2.4)	68	1.5 (0.0-7.9)	0.6 (0.5-0.8)
22	91	2.2 (0.3-7.7)	2.8 (2.4-3.2)	69	5.8 (1.6-14.2)	5.8 (5.1-6.6)
23	88	7.9 (3.2-15.7)	6.7 (5.5-8.0)	97	4.1 (1.1-10.2)	4.5 (3.7-5.4)
24	82	1.2 (0.0-6.6)	1.6 (1.1-2.4)	72	1.4 (0.0-7.4)	0.9 (0.6-1.3)
25	82	6.0 (2.0-13.6)	5.7 (4.7-6.7)	93	1.1 (0.0-5.8)	7.8 (-)
26	86	1.2 (0.0-6.3)	0.8 (0.5-1.1)	75	4.0 (0.8-11.2)	1.8 (1.4-2.4)
27	100	3.0 (0.6-8.5)	1.8 (1.4-2.5)	79	3.8 (0.8-10.7)	17.1 (16.5-17.7)
28	52	9.6 (3.2-21.0)	12.2 (10.2-14.5)	85	0 (0.0-4.2)	0.0 (-)
29	91	2.1 (0.3-7.7)	1.2 (1.0-1.5)	82	1.2 (0.0-6.6)	1.7 (1.3-2.3)
30	72	0.0 (0.0-5.0)	0.0 (-)	75	2.7 (0.3-9.3)	1.9 (1.4-2.5)
31	65	6.1 (1.7-15.0)	5.4 (0.4-7.2)	98	0.0 (0.0-3.7)	0.0 (-)
32	80	2.5 (0.3-8.7)	2.9 (2.0-4.1)	88	5.6 (1.9-12.8)	5.6 (4.7-6.6)
33	87	3.4 (0.7-9.7)	2.9 (2.3-3.6)	88	2.2 (0.3-8.0)	4.2 (3.9-4.4)
34	64	3.1 (0.4-10.8)	2.4 (2.0-2.8)	86	3.5 (0.7-9.9)	4.8 (3.9.0-5.7)
35	74	0.0 (0.0-4.9)	0.0 (-)	91	2.1 (0.3-7.7)	2.9 (2.1-4.0)

*Adjusted for survey design (clustering and household selection probability within PSU) and standardised for age and gender
