# Supplementary material for: Scabies prevalence after ivermectin-based mass drug administration for lymphatic filariasis, Samoa 2018–2019
Source: PLoS Negl Trop Dis. 2023 Aug 22;17(8):e0011549. doi: 10.1371/journal.pntd.0011549 (PMC10497159; doi:10.1371/journal.pntd.0011549)
Supplement: S3 Table — (PDF) [file pntd.0011549.s003.pdf]

S3 Table. Crude (univariate model) and adjusted (multivariable model) odds ratios for scabies prevalence (IACS B3, C1 and C2 classifications combined) in participants aged ≥5 years (eligible for ivermectin in 2018 MDA), Samoa, Surveys 1 and 2

	Sample size (N)	Scabies (n)	Prevalence* (95%CI)	Unadjusted Odds Ratio (95% CI)	Adjusted Odds Ratio (95% CI)^	Multivariable model *p* value
**Total participants**	4997	123	2.5 (2.3-2.8)			
**Survey**						
Survey 1	2389	41	1.7 (1.5-2.0)	1.0	1·0	-
Survey 2	2608	82	3.3 (2.9-3.8)	2.0 (1.7-2.3)	**2.2 (1.9-2.5)**	<0.001
**Region**						
Apia Urban Area	801	33	4.7 (4.0-5.5)	3.5 (2.5-4.8)	**3.4 (2.5-4.5)**	<0.001
North West Upolu	2024	46	2.3 (1.9-2.7)	1.7 (1.3-2.3)	**1.6 (1.2-2.2)**	0.004
Rest of Upolu	1195	26	2.4 (2.1-2.7)	1.8 (1.4-2.5)	**1.7 (1.2-2.3)**	0.001
Savai'i	977	18	1.3 (1.0-1.6)	1·0	1·0	-
**Number of residents in household**						
1-4	807	13	1.2 (0.9-1.5)	1·0	1·0	-
5-8	2064	44	2.1 (1.7-2.4)	2.0 (1.5-2.6)	**2.0 (1.5-2.6)**	<0.001
9-12	1184	33	2.9 (2.5-3.4)	2.9 (2.2-3.9)	**2.9 (2.2-3.9)**	<0.001
13-20	942	33	3.6 (3.1-4.3)	3.6 (2.7-4.9)	**3.6 (2.6-4.9)**	<0.001
**Gender**						
Male	2279	51	2.2 (1.9-2.5)	1.0	1.0	-
Female	2718	72	2.8 (2.5-3.2)	1.2 (1.0-1.4)	**1.2 (1.0[#]-1.4)**	0.023
**Age groups in years**						
5-15	1791	60	3.6 (3.2-4.1)	1.7 (1.5-1.9)	**1.6 (1.4-1.8)**	<0.001
≥16	3206	63	2.1 (1.8-2.3)	1·0	1·0	-
**At least one employed person screened in the household**						
Yes	2193	52	2.5 (2.1-3.0)	1·0	1·0	-
No	2804	71	2.6 (2.2-3.0)	1.0 (0.8-1.2)	**1.3 (1.0[#]-1.6)**	0.023
**Took ivermectin-based mass drug administration in 2018**						
Yes	4420	107	2.3 (2.1-2.6)	1·0	1·0	-
No	577	16	3.6 (2.8-4.7)	1.4 (1.1-1.8)	**1.2 (1.0[#]-1.5)**	0.02

*Adjusted for survey design (clustering and household selection probability within PSU) and standardized for age and gender

^Multivariable logistic regression model adjusted for survey design (sampling probability by PSU and household)

[#] Lower 95% CI >1.0, but rounded down to 1.0 when using one decimal place.
